# Maize leaf disease recognition using PRF-SVM integration: a breakthrough technique

**DOI:** 10.1038/s41598-024-60506-8

**Published:** 2024-05-03

**Authors:** Prabhnoor Bachhal, Vinay Kukreja, Sachin Ahuja, Umesh Kumar Lilhore, Sarita Simaiya, Anchit Bijalwan, Roobaea Alroobaea, Sultan Algarni

**Affiliations:** 1 Chitkara University Institute of Engineering and Technology, Chitkara University, Punjab, India; 2 University Institute of Engineering Chandigarh University Punjab, Mohali, India; 3 Arba Minch University, Arba Minch, Ethiopia; 4 Department of Computer Science, College of Computers and Information Technology, Taif University, P. O. Box 11099, 21944 Taif, Saudi Arabia; 5 Department of Information Systems, Faculty of Computing and Information Technology, King Abdulaziz University , 21589 Jeddah, Saudi Arabia; 6 School of Computing Science and Engineering, Galgotias University, Greater Noida, UP India

**Keywords:** Maize leaf diseases, Segmentation, Convolutional neural network, Classification, Fuzzy SVM, Biotechnology, Plant sciences

## Abstract

The difficulty of collecting maize leaf lesion characteristics in an environment that undergoes frequent changes, suffers varying illumination from lighting sources, and is influenced by a variety of other factors makes detecting diseases in maize leaves difficult. It is critical to monitor and identify plant leaf diseases during the initial growing period to take suitable preventative measures. In this work, we propose an automated maize leaf disease recognition system constructed using the PRF-SVM model. The PRFSVM model was constructed by combining three powerful components: PSPNet, ResNet50, and Fuzzy Support Vector Machine (Fuzzy SVM). The combination of PSPNet and ResNet50 not only assures that the model can capture delicate visual features but also allows for end-to-end training for smooth integration. Fuzzy SVM is included as a final classification layer to accommodate the inherent fuzziness and uncertainty in real-world image data. Five different maize crop diseases (common rust, southern rust, grey leaf spot, maydis leaf blight, and turcicum leaf blight along with healthy leaves) are selected from the Plant Village dataset for the algorithm’s evaluation. The average accuracy achieved using the proposed method is approximately 96.67%. The PRFSVM model achieves an average accuracy rating of 96.67% and a mAP value of 0.81, demonstrating the efficacy of our approach for detecting and classifying various forms of maize leaf diseases.

## Introduction

Many factors, including pollinator population declines, the effects of climate change, the spread of plant diseases, and a variety of other difficulties, pose serious concerns to food security. Plant diseases, in particular, imperil global food security, particularly for small-scale farmers who rely significantly on agriculture and successful harvests for a living. It is worth noting that smallholder farmers account for more than 80% of agricultural production in developing countries^1^ and reports show that more than 40% of crops are lost to pests and diseases^2^. Estimates show that by 2050, the global population will have surpassed 9.7 billion people, highlighting the critical need to ensure food security. To successfully respond to this challenge, rapid and precise plant disease detection tools are required^3,4^.

Maize, a major cereal crop, is grown all over the world. It has the highest global production of any grain crop, and hence plays a critical role in guaranteeing food security, the production of feedstock, and meeting the need for energy in a population that is rising worldwide^5^. Maize plays a significant part in the economy as a primary source of raw materials for a wide array of industrial products. It is important because it is a primary source of nutrition for a wide variety of organisms, including humans and animals. The maize crop is vulnerable to a wide variety of infectious illnesses as well as insect infestations. Diseases affecting maize leaves, in particular, cause productivity decreases and economic setbacks for farmers. Nevertheless, in spite of its high yield potential, the maize plant is susceptible to a wide range of illnesses, which results in a loss of 6% to 10% of its crop every year^6,7^. These maize diseases are caused mostly by viroids, bacteria and fungi. Discoloration, decay, scabbing, blight, necrosis, wilting, and distortions are all symptoms of infection that can be used to detect and identify foliar diseases in maize. The most frequent fungal foliar infections impacting maize agriculture are northern corn leaf blight (NLB), southern corn leaf blight (SLB), and grey leaf spot (GLS)^8^. Currently, it is essential to maintain maize productivity through accurate diagnosis of illnesses that affect maize leaf tissue, especially for farmers who lack specialised knowledge. The traditional method of identifying maize illnesses involves physical leaf inspection and is based on plant pathology knowledge as well as the expertise of agricultural specialists. The incorrect diagnosis of these disorders frequently leads to insufficient pesticide treatment, resulting in contamination and increased toxicity to maize. As a result, there is an urgent need for quick and precise technologies to monitor maize fields and control diseases.

**Figure 1:**
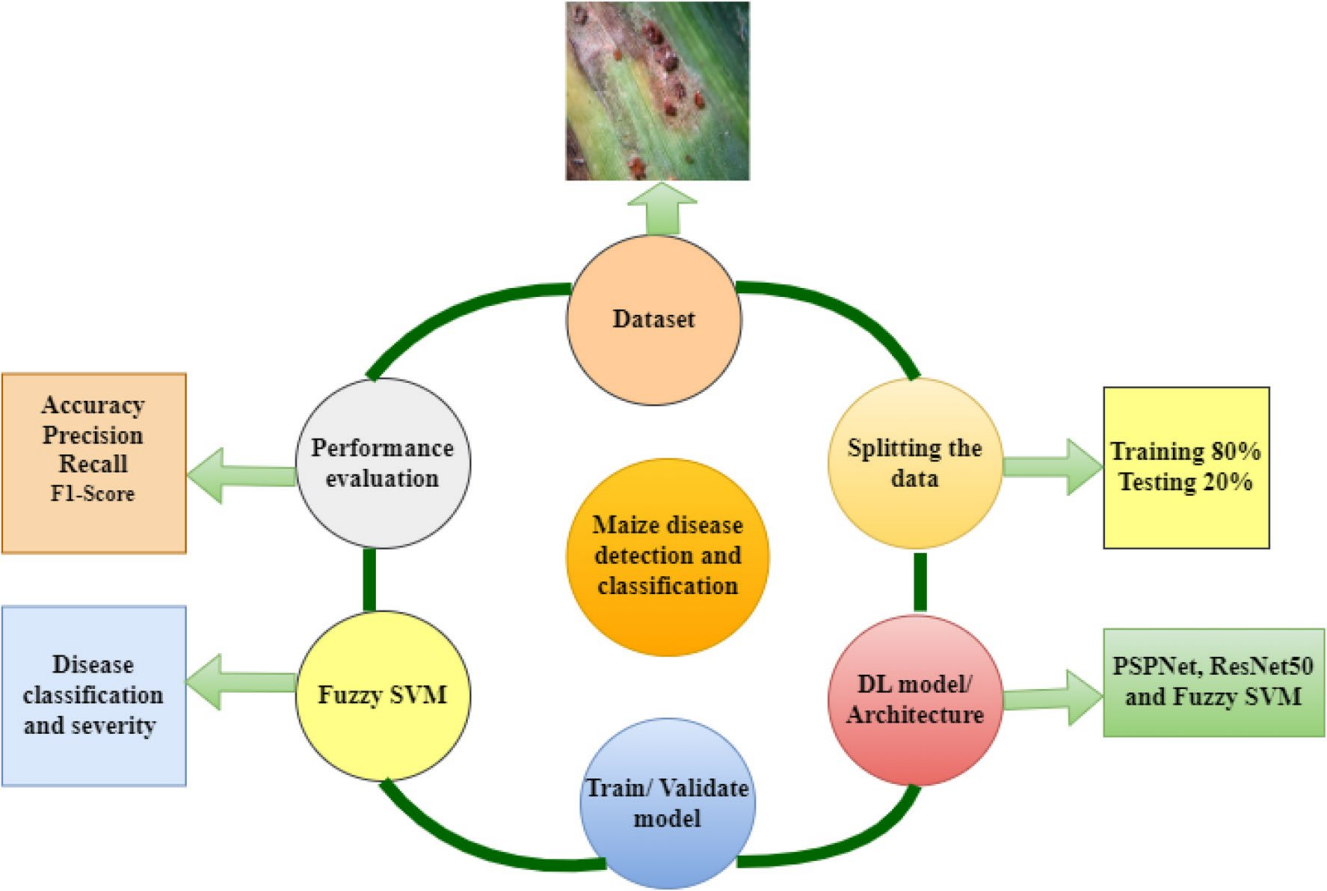
Maize leaf diseases classification strategy.

Machine learning (ML) and Deep learning (DL) are organized into layers within the wide domain of artificial intelligence, demonstrating the field's vastness. The advancement of Artificial Intelligence (AI) and the creation of image-processing tools creates potential for agricultural research to advance. Deep learning, a subset of machine learning, is currently a growing domain with numerous successful applications. Deep neural networks, which are an essential component of deep learning, excel in tasks such as feature extraction, pattern recognition, and data classification^9^. They have also proven their worth in a variety of areas, including agriculture, commerce, automotive, telecommunications, and networking. This achievement might be due to the use of techniques such as object detection and image categorization^10,11^. The traditional way of identifying plant diseases in agriculture relies on manual approaches that necessitate visual evaluation expertise. Following that, more comprehensive research is conducted in laboratories, which is both time-consuming and frequently out of reach for small-scale farmers. As a result, scientists have concentrated their efforts on automated and intelligent disease detection systems. These systems use artificial intelligence, machine learning, and deep learning techniques to provide novel solutions that have the potential to transform disease detection in agriculture. Deep learning algorithms are generally concerned with collecting features from images and then applying these features to classification or regression tasks, depending on the objectives.Although each deep learning algorithm extracts features in its own way, merging the collected features from various deep learning algorithms can result in better results. This is because the classifier obtains access to a broader range of descriptive information, which improves its learning capabilities. Figure 1 shows the maize leaf disease classification strategy.

This work presents a novel classification technique for accurately categorising digital images of maize leaves with grey leaf spots, common rust, southern rust, northern leaf blight, southern leaf blight, and healthy leaves.

### Problem statement and contribution

Maize crop infections are recognized using computer vision and image processing in four stages: preprocessing original data, segmenting contaminated regions, extracting features, and classifying infection types. Our endeavor concentrated on extracting and fusing multi-layer characteristics. The automated recognition system faces challenges in accurately identifying crops due to similarities in color and texture, changes in symptom shapes for disease regions, and variation in illumination and background. The major contribution of this study is as follows:

- The current study improves classification accuracy by recognizing several fungal maize diseases through the integration of PSPNet, ResNet-50 and fuzzy SVM model.
- The fundamental application of PRFSVM model shows the machine learning flexibility for multiclass maize diseases classification and helps accurate choice-making in real-time farming environments that will potentially help to reduce the yield quality losses.
- The proposed model helps to minimize maize yield quality, which helps to maintain sustainable agricultural practices.

### Highlights of this study

- To suggest the PSPNet and RESNet50 for semantic segmentation and feature extraction tasks and discuss how combining the capabilities of these two models increases semantic segmentation accuracy and robustness.
- Describe the context of PSPNet's pyramid pooling module is used to extract the sub-images at different scales in different orientations. Once the sub-images of each leaf have been extracted, selective attributes in the form of their features have been extracted by RESNet50's pre-trained model
- Derive the Fuzzy rules along with the SVM model that can easily determine the classification of each maize leaf disease along with its severity.
- In order to illustrate the effectiveness of our method, we subjected it to extensive evaluation on a database that could be accessed online. The findings demonstrate that the strategy that was proposed is effective even when subjected to challenging situations such as the presence of a crowded background, variations in lighting, and distortions.

### Paper organization

The framework that follows can be found in the manuscript: In Section Related work, we investigate existing research pertaining to the identification of plant diseases, focusing specifically on maize crop diseases. The selected approach as well as the specific design of the suggested framework are broken down and discussed in Section Materials and methods. The details of the experimental results that were chosen, along with an analysis and a discussion, are presented in Section "Experiment results, analysis and discussion". In the final step, we brought our work to a close and presented some recommendations for the future in Section Conclusions and future directions.

## Related work

Scientists have developed a number of ways for locating, classifying, and computing the associated properties of diverse plant disorders. As a result, Machine learning (ML) and Deep learning (DL)techniques are widely used by researchers. Within this section, we examined the current literature on the classification of maize leaf anomalies.

Zhang et al.^12^ classified the various maize leaf diseases using a special dataset using SVM classifiers. The RBF kernel function has the highest accuracy in various sorts of categorization, with an average recognition rate of 89.6% for category 5 of selected disorders. The Sigmoid kernel function ranks second, with an average of 83.2%. Zhang et al.^13^ used their study to introduce the GA-SVM algorithm, which outperforms the classic SVM method. Genetic algorithms are used to determine the penalty factor and kernel function automatically. Their study achieves an amazing classification score of 90.25% by using a specialized dataset. Furthermore, Zhang et al.^14^ proposed a leaf recognition system based on plant illnesses. Following the segmentation of the affected area, the illness feature vector is extracted. These obtained attributes are then sent into the K-nearest-neighbor classifier to identify plant diseases. The experimental findings support the effectiveness of the proposed approach, delivering a 90.30% accuracy rate. Xu et al.^15^ compiled a database of seven different forms of maize leaf diseases. This database contains 516 photographs taken in the field under natural lighting conditions. Experiments in the database demonstrate that three types of classifiers have excellent recognition effects, with recognition rates of more than 85%, indicating that the three features are acceptable for recognising maize leaf diseases. The proposed method has a recognition rate of 94.71% and it takes roughly 1.382 s to identify an image. Qi et al.^16^ presented a work to identify disease spots, colour, texture, and invariant moments, an automatic threshold approach in RG grey space was used. To identify several prevalent illnesses on maize leaves, the principal component analysis method and the support vector machine were utilised. The experimental findings revealed that the total recognition accuracy was 90.74%.

The analysis that was performed above demonstrates that despite the fact that a variety of ML techniques for classifying maize leaf diseases have been proposed in published research, Because of their limited ability to distinguish between different forms of data, ML methods are unable to properly capture the structural information of the samples. The robustness and superior memory capacity of the DL methods have persuaded researchers will put them to the test in the realm of classifying maize plant diseases.

Haque et al.^17^ proposed a strategy based on deep convolutional neural networks for automatically identifying digital photos of maize crop illnesses in addition to photographs of healthy maize leaf tissue. A total of 5939 photos of maize crops taken in the field were collected from experimental fields situated in one, two and three different maize growing zones. Three network topologies were modelled on the maize dataset for the 'Inception-v3'network. In these architectures, they have used baseline learning to train all of the computational layers using their maize dataset. According to the experimental results, Inception-v3_GAP had the greatest accuracy of 95.99% in a separate test dataset. The Inception-v3_GAP model proved effective at learning key features from disease symptoms and predicting accurate class levels in previously unknown data.Subramanian et al.^18^ used a collection of 18,888 photos of healthy and diseased leaves to classify three common maize leaf diseases using pre-trained ResNet50, Xception, VGG16 and InceptionV3models. They classified three common maize leaf diseases using pre-trained InceptionV3, VGG16, ResNet50, and Xception models. Bayesian optimisation is used to find the best hyperparameter values, while image augmentation is used to improve the model's generalizability. According to the results,all trained models demonstrated higher than 93% accuracy in diagnosing maize leaf diseases.

Baldota et al.^19^ used highly linked convolutional neural networks on the PlantVillage dataset. DenseNet121 outperformed by achieving an accuracy of 98.45% on the test set while requiring less storage space and training time. Ahila et al.^20^ proposed a deep convolutional neural network (CNN)-based architecture (modified LeNet). This experiment was conducted using maize leaf images from the PlantVillage dataset. The proposed CNN is trained to recognize four unique classes: three diseased and one healthy. The accuracy of the proposed model is 97.89%. Simulation results in maize leaf disease classification show the potential efficiency of the proposed strategy. Jiangchuan et al.^21^ adopted an EfficientNet-based transfer learning approach. The EfficientNet experiment used the original training, but the control group also included additional training comparison trials such as VGG-16, Inception-V3and Resnet-50. With a maximum recognition accuracy of 98.52%, it outperforms other networks in agricultural production applications. Zhang et al.^22^ proposed the improved deep learning-based GoogleNet and Cifar10 models for leaf disease recognition in this research. Two improved models were developed for training and evaluation of 9 different types of corn leaf images by adjusting the parameters, changing the fusion composition, adding a deletion operation and a modified linear unit function, and by reducing the number of classifiers. When detecting eight types of corn leaf diseases, the GoogleNet model has the highest average detection accuracy of 98.9%, and the Cifar10 model achieves an average accuracy of 98.8%.

Mingjie et al.^6^ proposed a method for identifying maize leaf diseases based on image enhancement using DMS-Robust Alexnet.Batch normalisation is carried out in order to stop the network from becoming over-fit to the data while simultaneously improving the model's robustness. The DMS-Robust Alexnet was created with the goal of recognition and classification in mind, and its recognition accuracy can reach up to 98.62%. Hassan et al.^23^ proposed approach extracts deep information from maize plant images used two pre-trained convolutional neural networks (CNNs), DenseNet121 and EfficientNetB0. The results of this study have been compared with two pre-trained CNN models, ResNet152 and InceptionV3. Both have more parameters than the proposed model and require more processing resources. When compared to ResNet152 and InceptionV3, which obtained classification accuracies of 98.37 and 96.26%, respectively, the suggested model achieves a classification accuracy of 98.56%. Zeng et al.^24^ suggested a model that replaces the ResNet-50 backbone network's 3 × 3 convolution kernel with the Select Kernel-Point-Swish_B (SKPS) module. This SKPS module is an improved building block derived from the selected core unit (SK). In addition, the standard ReLU activation function has been replaced by the Swish_B activation function. These changes are intended to improve the model's ability to extract characteristics from damaged leaves with small spots and irregular forms, hence improving its performance. The experimental results show that, when compared to machine learning models and deep neural network models, the suggested approach is significantly better at recognising maize leaf illnesses in natural scene photos. It achieves an average identification accuracy of 92.9%, a 6% improvement over the SKNet-50 model's performance.

Ahmad et al.^25^ evaluated several deep learning-based image categorization models by training them with diverse dataset combinations. They used transfer learning with five different pre-trained deep neural network architectures for these experiments: DenseNet169, InceptionV3, ResNet50, VGG16and Xception. These models were used in four different experiments. Following the training phase, they evaluated each deep learning model's generalization capability by using photos of different maize illnesses from diverse datasets as testing data. The DenseNet169 model got the highest level of generalization accuracy (81.60%) when trained using images with red, green, blue, and alpha (RGBA) channels from the CD&S maize disease dataset with backgrounds removed. Ying et al.^26^ proposed the use of Convolutional Neural Networks (CNN) combining multi-scale features to diagnose corn diseases. They have made many changes to the one-stage plant disease network YOLOv5s. To begin, they incorporated the Coordinate Attention (CA) attention module, as well as a critical feature weight, to increase the amount of meaningful information in the feature map. Furthermore, the researchers improved the Spatial Pyramid Pooling (SSP) module by implementing data augmentation algorithms to avoid feature information loss. In terms of average accuracy, the experimental findings clearly show that the MFF-CNN outperforms currently used approaches.

Hieu et al.^27^ used the ‘Simple Linear Iterative Clustering’ (SLIC) segmentation approach to analyse maize leaf pictures from both the CD&S and PlantVillage datasets. This method was used to create super-pixels, which are groups of pixels that represent different areas of interest on a maize leaf. VGG16, DenseNet121, ResNet50, InceptionV3, and Xception are five pre-trained deep learning models that detect abnormal regions that correspond to five superpixel classes. This was achieved after the spatial proximity parameter (sigma) of the SLIC segmentation was set to five. After training the DenseNet121 model, the overall testing accuracy reached 97.77%. In this paper^28^, the AlexNet model was used to study the speedy and precise identification of leaf diseases in maize plants. The model attained an astounding 99.16% accuracy rate after several iterations, including 25, 50, 75, and 100.Chenghai et al.^29^ presented DISE-Net, an innovative deep-learning architecture. They created an enhanced Inception module to replace the standard inception module in order to improve the performance of multi-scale feature extraction. To complement their research, they have created a collection of 1268 photos of four disease grades and healthy maize leaves, with an emphasis on small leaf spots. The results from comparative studies show that DISE-Net achieves an amazing test accuracy of 97.12%, outperforming traditional deep learning models.

This study^30^ offers a new network design called MAM-IncNet. The authors replace the old SSD convolutional layers with optimised Inception modules (M-Inception), and the pre-trained VGG16 serves as the backbone network. In addition, a hybrid attention mechanism including channel-wise and spatial attention is implemented. The proposed technique achieved a recall rate of 81.44%. The author^31^ investigated deep learning approaches and developed a convolutional ensemble network to improve the model's ability to detect minute plant lesion features. We used the ensemble learning method to aggregate three lightweight CNNs, SE-MobileNet, Mobile-DANet, and MobileNet V2, to construct a new network named Es-MbNet, which can recognise plant disease kinds. The proposed approach achieved 99.61% accuracy. This research ^31^ presents a novel method for detecting plant leaf diseases. The method is broken into two steps: picture segmentation and image classification. First, a hybrid segmentation algorithm based on hue, saturation, and intensity, as well as LAB, is proposed and applied to segment disease symptoms from plant disease photos. The validation accuracy obtained with this approach was roughly 15.51% greater than that of the conventional method.

The author's^32^ use of new sensors and imaging techniques considerably increased the efficiency and accuracy of the ANN model. The procedure is largely dependent on the quality of data sets and the algorithm used to process them. This study examines the benefits of using artificial neural networks to achieve optimal or near-optimal solutions. In this research^33^, the authors investigate one of the optimisation methods known as the Bacterial Foraging Optimisation Algorithm (BFOA). This method is then used to set the weights for the Artificial Neural Network (ANN) that is utilised for image segmentation. Lastly, when the findings contrast to other approaches, the BFO-ANN technique proves to be more efficient. The author’s^34^ suggested a machine learning method for segmenting plant diseases from leaf photos that employ the Radial Basis Function Neural Network (RBFNN). The photos used in this study were obtained from the IPM agriculture database repository. The results of the experiments show that the suggested RBFNN produces higher segmentation accuracy than the other techniques. This paper^35^ proposed a computer vision methodology for defining the need and automating a disease detection systems. A Hybrid Neural Network with Superpixel Clustering is proposed for disease region segmentation. Different algorithms are used to analyse the colour, shape, and texture aspects. In this research^36^, the authors conducted a survey of the number of articles that use computer vision and soft computing methods to identify and classify diseases from plant leaves. The objective is to convey the cutting-edge principles, applications, and theories behind digital image processing and soft computing approaches. The different results have been addressed separately.

The researcher^37^ investigates the transformative powers of Deep Learning (DL) models, with a particular emphasis on Convolutional Neural Networks (CNNs) and MobileNet architectures for the early and precise detection of plant disease. Authors expanded their investigation by introducing eXplainable Artificial Intelligence (XAI) via GradCAM, which clarified how decisions are made of these models and provided a visual interpretation of illness signs in plant photos. After thorough testing, the CNN model achieved an accuracy of 89%, precision and recall of 96%, and an F1-score of 96%. In contrast, the MobileNet architecture achieved an accuracy of 96% but had somewhat lower precision, recall, and F1-scores of 90%, 89%, and 89%, respectively. The authors^38^ identify the key factors that provide initial data on the grapevine's health. This paper presents an overview of the sensor network's design and components. To identify and predict grapevine illnesses, sensor data is analysed using Machine Learning (ML) algorithms and linked with results obtained using classical approaches. The disease occurrence results are reported, as well as the environmental parameters that influence them.

## Materials and methods

**Figure 2:**
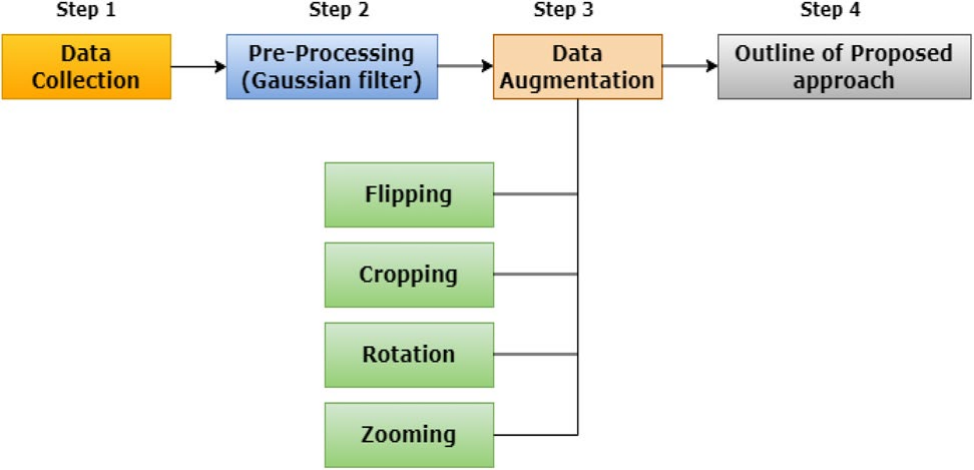
Overflow of Maize leaf diseases identification methods.

CNNs' remarkable performance in image classification and object recognition tests prompted academics to study the networks' potential for semantic segmentation tasks. Since then, numerous architectural ideas have been published in academic journals, demonstrating promising results in a variety of fields of expertise. We chose the PSPNet semantic segmentation architecture from a pool of accessible options for this project. Several CNN architectures have arisen, each with its own set of properties. Regardless of these variances, they all share a common goal: to improve accuracy while simultaneously reducing model complexity. Certain architectures deliver great performance across a wide range of applications. GoogleNet^39^, VGG16^40^, ResNet50^41^, and AlexNet^42^ are some of the popular options for addressing the plant disease categorization challenge. Following that, we present the suggested architecture, which is divided into three stages: a semantic segmentation phase learned on the segmentation dataset, feature extraction, and classification phase trained on the symptoms dataset. The initial model's segmented symptoms are then independently processed and categorized within the second model. As a result, this method allows for the identification of many symptoms as well as the precise assessment of their severity levels. The overflow of maize leaf disease identification methods is described in Fig. 2.

### Data collection and pre-processing

**Figure 3:**
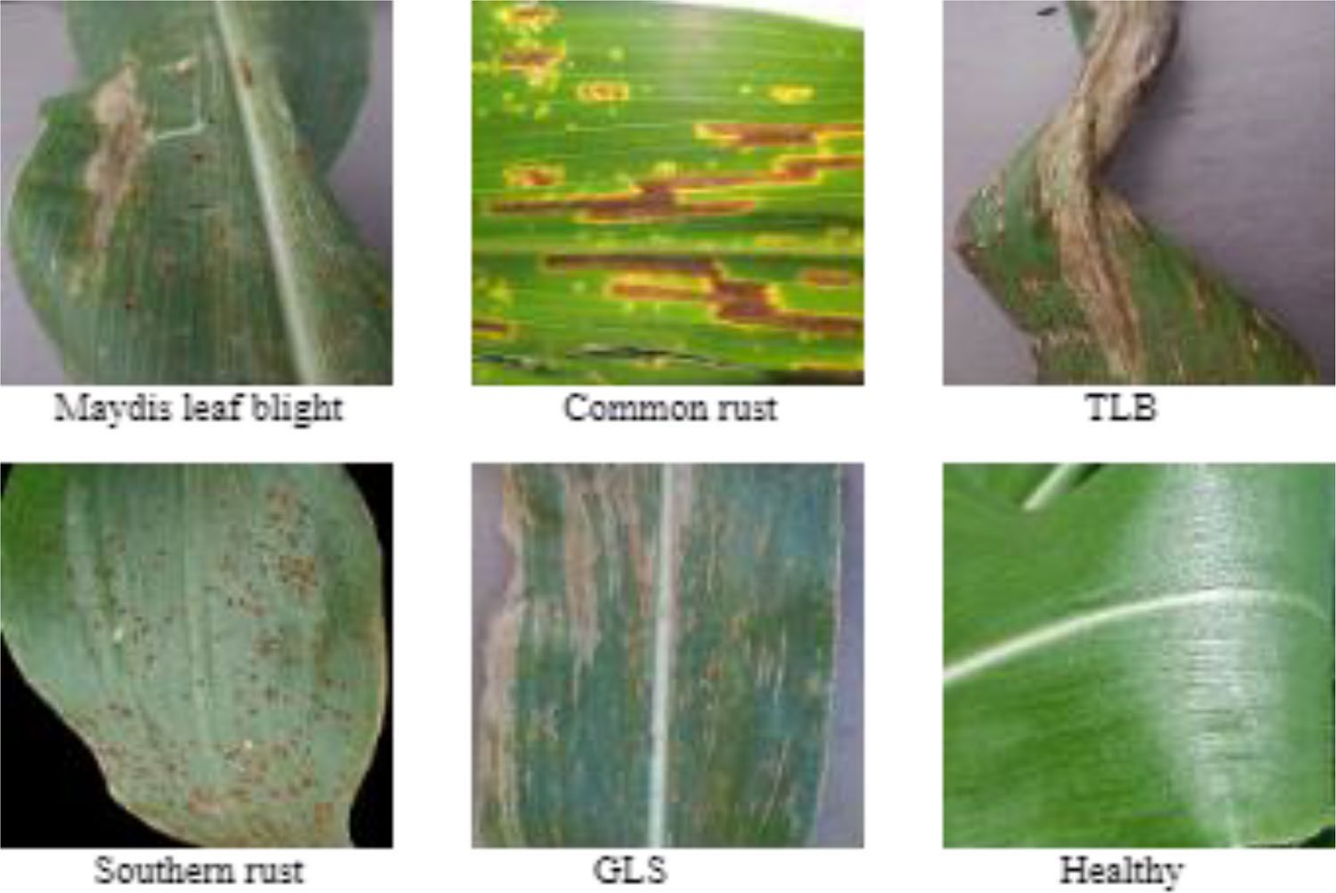
Samples of pre-processed maize leaf diseases.

This paper used maize disease images from the PlantVillage dataset in this work, which are distinguished by their uniform backgrounds in images demonstrating various maize diseases. First, the dataset is separated into two classes: diseased and healthy. The collected images are classed and tagged. The annotated images were used to train the PSPNet model, while the categorized class images were utilized to categorize the various maize illnesses. All of the photographs were collected in both JPEG and PNG formats. The dataset contains images of several diseases affecting maize plants, allowing for the development and testing of machine-learning models for identifying and diagnosing these diseases in maize crops. The dataset included 1532 images displaying Common Rust, 1430 images depicting Southern Rust, 1139 images depicting Grey Leaf Spot (GLS), 574 images depicting Maydis leaf blight (MLB), and 456 images depicting Turcicum Leaf Blight (TLB) leaf disease. There were also 1587 images in the dataset that depicted the healthy state of maize leaves. The leaf image is resized 224 × 224x3 in this work and then utilised to test the performance of the suggested model. Figure 3 shows the samples of pre-processed images.

**Figure 4:**
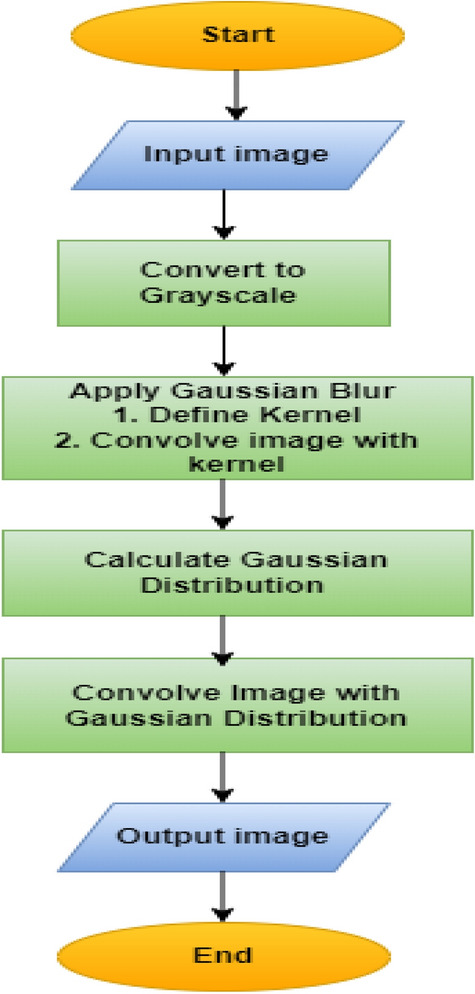
Perform image pre-processing through Gaussian filter.

In this research, the Gaussian blurring technique is used for noise reduction. It convolves the input image with a Gaussian kernel, averaging pixel values in local neighborhoods. This reduces the impact of random noise and slight visual fluctuations, resulting in cleaner input data for the neural network. The Gaussian blurring technique is often used in conjunction with a Gaussian kernel in a convolution procedure as shown in Fig. 4. The Gaussian kernel functions in two dimensions. Gaussian blurring can be calculated as follows:

1 $${G}_{blurred}\left(u,v\right)= \sum_{i=-\infty }^{\infty }\sum_{j=-\infty }^{\infty }B\left(u+i,v+j\right). A(i,j)$$

where:${G}_{blurred}\left(u,v\right)$ is the pixel value in the blurred image at coordinates (u,v), B(u + i,v + j) indicates the pixel value in the original input image at coordinates (u + i, v + j), A(i,j) is the Gaussian kernel value at coordinates (i, j).

A(i,j) is the Gaussian kernel defined as:

2 $$A\left(i,j\right)=\frac{1}{2\pi {\sigma }^{2}}.e\frac{-{i}^{2}+{j}^{2}}{2{\sigma }^{2}}$$

where: $\sigma$ is the standard deviation of the gaussian distribution.

By smoothing out minor changes in pixel values, Gaussian blurring helps to reduce noise, resulting in cleaner and more accurate data. To improve the interpretability of learnt features, Gaussian blurring can be used to intermediate feature maps in deep neural networks. Smoothing these feature maps could help you visualise and comprehend the network's representations.

#### Challenges of data preprocessing

- The internet source dataset includes irrelevant or misleading information, such as watermarks that are logos, or text, which can lead to confusion about the authenticity of the photos.
- The noise in visual analysis can drastically reduce efficiency and efficacy. Electronic equipment and unique lighting effects cause this type of interruption. The method of prediction may be prevented if an image of a leaf contains multiple types of noise, such as Gaussian noise, pulse noise, salt and pepper noise, and so on^13^.
- Determining the optimum image size is a tough part of this research investigation. Although every bit of visual data that comprises a large image may have a considerable impact on the viewer, the combined effect of all of that information can be quite enormous^29^.

### Data augmentation

Data augmentation is an essential approach in Machine learning (ML) and Deep Learning (DL) that is used to increase the size and diversity of training datasets. It requires making several changes to the original data and generating new samples with minor changes while preserving the underlying patterns and attributes. In the context of image data, these transformations can include rotations, flips, scaling, cropping, changes in brightness or contrast, and more. The model gets exposed to a greater range of variables as the dataset is expanded, making it more robust and capable of generalising to previously unknown data. This method is particularly effective when the original dataset is tiny, since it prevents overfitting while also improving the overall performance and dependability of machine learning models.

1. Flipping: Flipping is a computer vision and image processing technique that involves reversing the horizontal or vertical pixel values of an image, resulting in a mirror image. By flipping the original image vertically or horizontally, this filter copies it.
2. Zooming: The zooming is done at random, with the degree of zoom calculated independently for each image and limited to 10%.
3. Cropping: By selecting alternative cropping windows or methods at random for each iteration, the dataset's diversity was improved.
4. Rotation: Images are rotated at random by an angle within a predetermined range. For full rotations, the range of rotation angles is limited to − 90 to + 90 degrees in this research.

The unequal number of samples has an impact on model recognition accuracy. As a result, four general methods are used to augment a small number of sample data: random rotation, flipping, zooming and cropping. To avoid severe deformation of the converted images, the displacement of the key points in the point of view transformation has been limited to less than 10% of the image's side length. The size of the limited number of sample data is raised by four times. Through data augmentation, the dataset increases with 8,443 images. The dataset was divided into two parts: 80% for training and 20% for testing the model's performance. From the training subset, a validation split of 20% of the training data was taken. To learn the intricate aspects of the images, the model is fed the training subset. The validation subset, on the other hand, is separated from the training subset data and is used to monitor the model's performance. This is accomplished by feeding it validation information after every training epoch and analyzing its performance. Following the training phase, the test subset is used to evaluate the model's overall performance on data it has never seen before.

### Outline of PRFSVM model

The PRFSVM model combines three fundamental components for image analysis viz. PSPNet, ResNet50, and Fuzzy SVM. This model is intended to solve several aspects of image analysis, such as image segmentation, classification, and uncertainty handling using Fuzzy SVM approaches. It means that PRFSVM is about more than just integrating these three components; it's about providing a comprehensive solution for a wide range of image analysis.

#### Fundamental architecture of PSPNet

**Figure 5:**
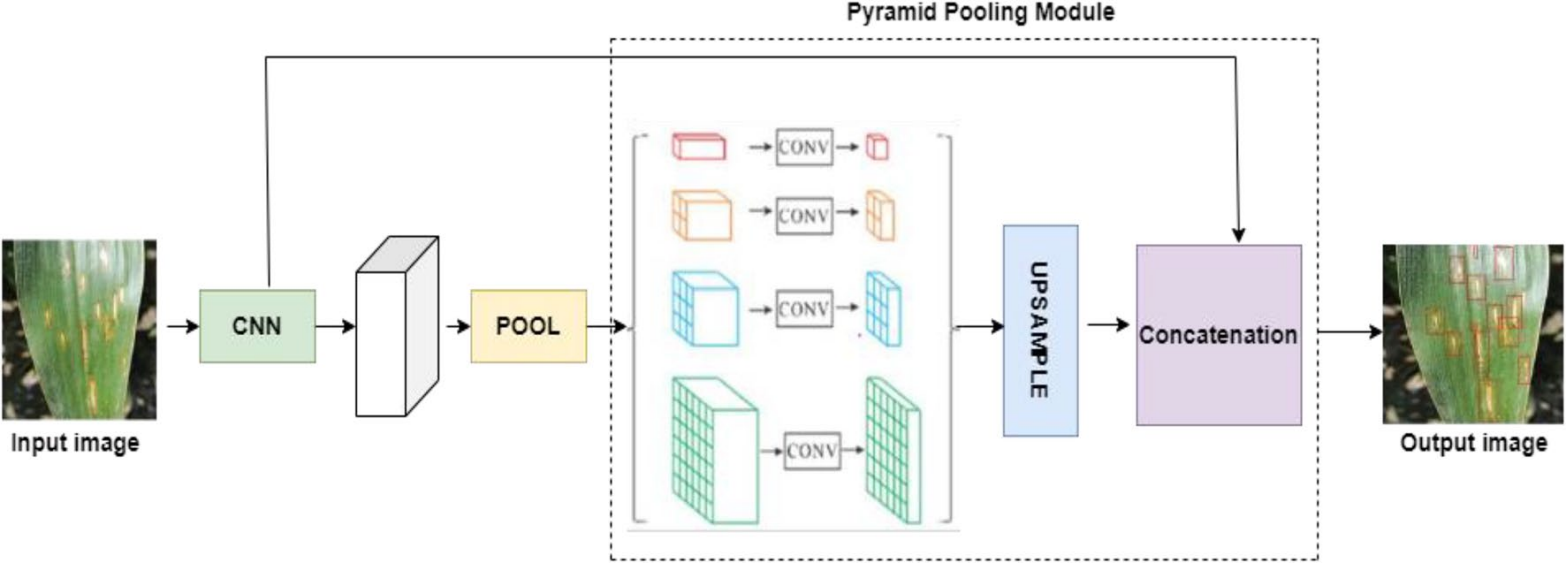
Architecture of PSPNet.

In this study, we have employed well-established semantic segmentation architectures found in existing literature: PSPNet. The Pyramid Scene Parsing Network (PSPNet) architecture, as introduced by Zhao et al.^43^ was used. PSPNet is a semantic segmentation network specifically intended for segmenting complex scenarios in which complete global context information is critical for identifying related items. PSPNet, or Pyramid Scene Parsing Network, employs a multi-step strategy to successfully segment leaf diseases^44,45^. It starts with a maize leaf image as input and then uses a number of convolutional layers to extract complex details from the image. These traits capture information ranging from fine surface aspects of the leaf to broader contextual information. PSPNet, crucially, has a pyramid pooling module that collects features at different spatial scales. This enables the network to grasp both local and global context inside the image, which is critical in distinguishing between disease-affected and healthy regions. Following contextual enrichment, the network performs semantic segmentation, assigning a semantic label to each pixel in the image, indicating whether it refers to a healthy or diseased area. Subsequent post-processing techniques may improve the accuracy of the segmentation results. The end result is a segmented image in which colours or labels distinguish between distinct groups, such as healthy and diseased maize leaf parts. PSPNet's technique is effective for segmenting leaf diseases, which aids in agricultural disease diagnosis and management. Figure 5 shows the architecture of PSPNet.

Here's a simplified illustration of the PSPNet architecture, with a focus on the pyramid pooling module:

To retrieve the output of the pyramid pooling module PM, run the input image 'I' through the PSPNet model 'PN'.

3 $${\text{PM}}\left( {\text{I}} \right) \, = {\text{ PN}}\left( {\text{I}} \right)$$

The pyramid pooling module 'PM' gathers context information at many scales by operating on the output of the preceding levels. PSPNet typically divides the feature map into many regions at various scales (for example, 1 × 1, 2 × 2, 3 × 3, and 6 × 6). Average pooling is used to generate a context vector for each region. The context vectors are then concatenated and upsampled to the original resolution. Context information from several scales is concatenated in the feature map.

The pyramid pooling module's concatenated feature map is then fed through further convolutional layers and softmax activation to obtain the final semantic segmentation map.

4 $${\text{PD }} = \, \left\{ {{\text{softmax}}} \right\}(\left\{ {{\text{conv}}} \right\}\left( {{\text{PM}}\left( {\text{I}} \right)} \right)$$

where 'PD' is the output tensor indicating the probability distribution of each pixel's membership in several semantic classes.

#### Feature pyramid network

**Figure 6:**
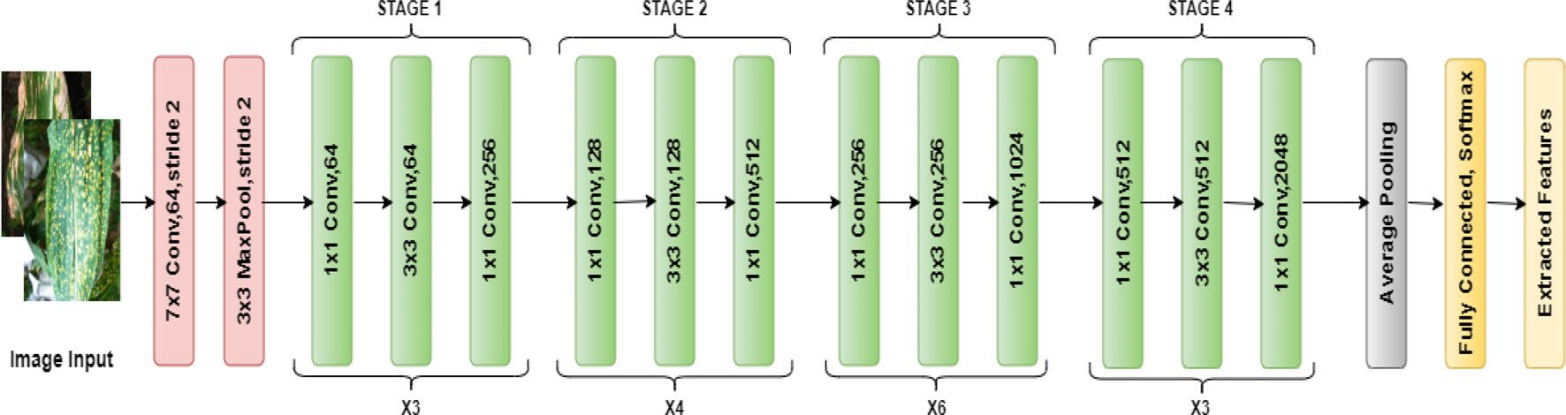
Layering architecture of RESNet50.

ResNet50, an abbreviation for Residual Network with 50 layers, is a ground-breaking deep convolutional neural network architecture that has considerably advanced the field of computer vision and image recognition. ResNet-50, introduced in 2015 by Kaiming He et al. ^46^, is unique in its capacity to address the vanishing gradient problem, a long-standing challenge in deep neural networks. The duo operation of "7 × 7 conv 64, stride 2" followed by "3 × 3 max-pooling, stride 2" at the beginning of the ResNet-50 architecture is a critical milestone in picture feature extraction. The operation "7 × 7 conv 64, stride 2" utilizes a 2D convolutional layer with a 7 × 7 kernel and 64 filters, with a stride of 2. This first convolutional layer serves a dual purpose by applying filters to the input image, capturing crucial visual elements while also lowering the spatial dimensions of the feature maps. Following that, the "3 × 3 max-pooling, stride 2" operation refines the feature extraction procedure even further. It down-samples the feature maps by using a 3 × 3 pooling window with a stride of 2, retaining the most prominent features while removing extraneous information. This gradual reduction in spatial dimensions is an important part of ResNet50's architecture, as it allows the network to learn increasingly abstract and sophisticated properties in succeeding layers. Figure 6 shows the layering architecture of ResNet50 and Table 1 shows the Layers parameters of ResNet50.

**Table 1:** Layers parameters of ResNet50.

Layers	Size dimensions	Stride/ Filter
Conv 1	64*256*256	2*2
Conv 2	32*128*128	2*2
Conv 3	16*64*64	2*2
Conv 4	8*32*32	2*2
Conv 5	4*16*16	2*2
Conv 6	4*8*8	2*2
Conv 7	4*8*8	2*2

Here's a simplified representation of the ResNet-50 feature extraction process:

Deviations:

- Image input: I with dimensions (Ht, Wd, Ch) (height, width, and channel count).
- ResNet-50 model: RM with many layers.
- FEL is the ResNet-50 model's feature extraction layer.

The feature extraction procedure is illustrated below:

1. Passing Forward Network:

- To obtain the feature extraction layer FEL output,run the input image ‘I’ through the ResNet-50 model RM.
  5 $$\left[ {{\text{FEL}}\left( {\text{I}} \right) \, = {\text{ RM}}\left( {\text{I}} \right)} \right]$$

2. Extraction of features:

- The output FEL(I) at layer (FEL) will have spatial dimensions Ht, Wd, and Ch, where (Ht' < Ht) and (Wd' < Wd) are high-level features learned by the network for the given input image.

3. Representation of Characteristics:

- Flatten the feature map into a 1D vector after obtaining (FEL(I).
  6 $$[{\text{FEL}}\left\{ {{\text{text}}\left\{ {{\text{flat}}} \right\}} \right\}\left( {\text{I}} \right) \, = {\text{ text}}\left\{ {{\text{flatten}}} \right\}\left( {{\text{FEL}}\left( {\text{I}} \right)} \right]$$
- To minimise the spatial dimensions while retaining key properties, use pooling techniques such as average pooling or max pooling with the Eq. (7).
  7 $$[{\text{FEL}}\left\{ {{\text{text}}\left\{ {{\text{pooled}}} \right\}} \right\}\left( {\text{I}} \right) \, = {\text{ text}}\left\{ {{\text{pooling}}} \right\}\left( {{\text{FEL}}\left( {\text{I}} \right)} \right]$$

#### Multi-class classification network model

Fuzzy Support Vector Machines (Fuzzy SVM) are employed in determining the severity of maize leaf diseases, especially when disease severity labels are not strictly binary but include a range of severity levels. Traditional SVMs are incapable of dealing with nuanced and inaccurate severity evaluations. Fuzzy SVM, on the other hand, adds fuzzy logic to the SVM framework, allowing for the representation of uncertainty in severity labels. This is useful in the context of maize leaf diseases, where disease severity might range from mild to severe in different cases. Fuzzy SVM assigns membership values to each severity level, capturing a sample's degree of belonging to various classes or severity categories.

Fuzzy SVM uses fuzzy membership functions to indicate the degree to which data points belong to distinct classes ^47^. The Gaussian membership function, which is frequently used in Fuzzy SVM, assigns a membership value to each data point in each class and has the following Eq. (8):

8 $${w}_{ij}\left(x\right)=e\left(\frac{-{\left|{x}^{-x}ij\right|}^{2}}{2{\sigma }_{i}^{2}}\right)$$

In this Eq. (8), the degree to which data point x belongs to class i is denoted by wij(x). It is computed by taking the Euclidean distance between x and the mean ${w}_{ij}$ of the Gaussian function for class i, scaled by the spread parameter ${\sigma }_{i}$.

Fuzzy SVM seeks a hyperplane that maximizes the margin between classes while taking into account fuzzy memberships and slack variables ($\varepsilon$ ij):

9 $${V}_{i}\left(w{x}_{i}+c\right)\ge 1-{\xi }_{i\dot{j}}$$

here $${\xi }_{i\dot{j}}\ge 0$$

In this modified objective function, w denotes the hyperplane's weight vector, and c the bias term. The fuzzy slack variables ( ${\xi }_{i\dot{j}}$ ) are introduced to allow certain data points to fall inside the margin of error or even be misclassified, reflecting the data's fuzzy character.

Fuzzy SVM's decision function uses fuzzy memberships and dual variables (${\alpha }_{ij}$) obtained during optimisation to forecast new data points in Eq. 10.

10 $$f\left(x\right)=\sum_{i=1}^{G}\sum_{j=1}^{H}{\alpha }_{ij}{y}_{i}{u}_{ij}\left(x\right)\left(wx+c\right)$$

Here,${\alpha }_{ij}$ denotes the dual variables determined throughout the optimisation procedure, and ${y}_{i}$ denotes the class labels. The decision function calculates a weighted sum of fuzzy memberships for each class. The dual variables (${\alpha }_{ij}$) indicate the relevance of each data item and its membership in class i. The resulting value aids in classifying fresh data items based on their fuzzy affiliations.

#### Advantages of FSVM

- The purpose of fuzzy membership is to lessen the impact of noise or outliers, and various fuzzy membership functions have varying effects on various categorization classes^13^.
- The aim of Support Vector Machines (SVM) is to find the best hyper plane to partition the feature space while maximizing the classification margin^47^.
- Every training sample can have a weight estimated by FSVM. To mitigate the effects of imbalanced datasets, FSVM steers clear of some low-weight samples, or noise samples, when building the classification hyper plane^47^.

### Ethical approval and consent to participate

No ethical approval is required, and the authors consent to participate in the paper.

### Consent for publication

Authors provide support for publication.

## General outline of the proposed model

**Figure 7:**
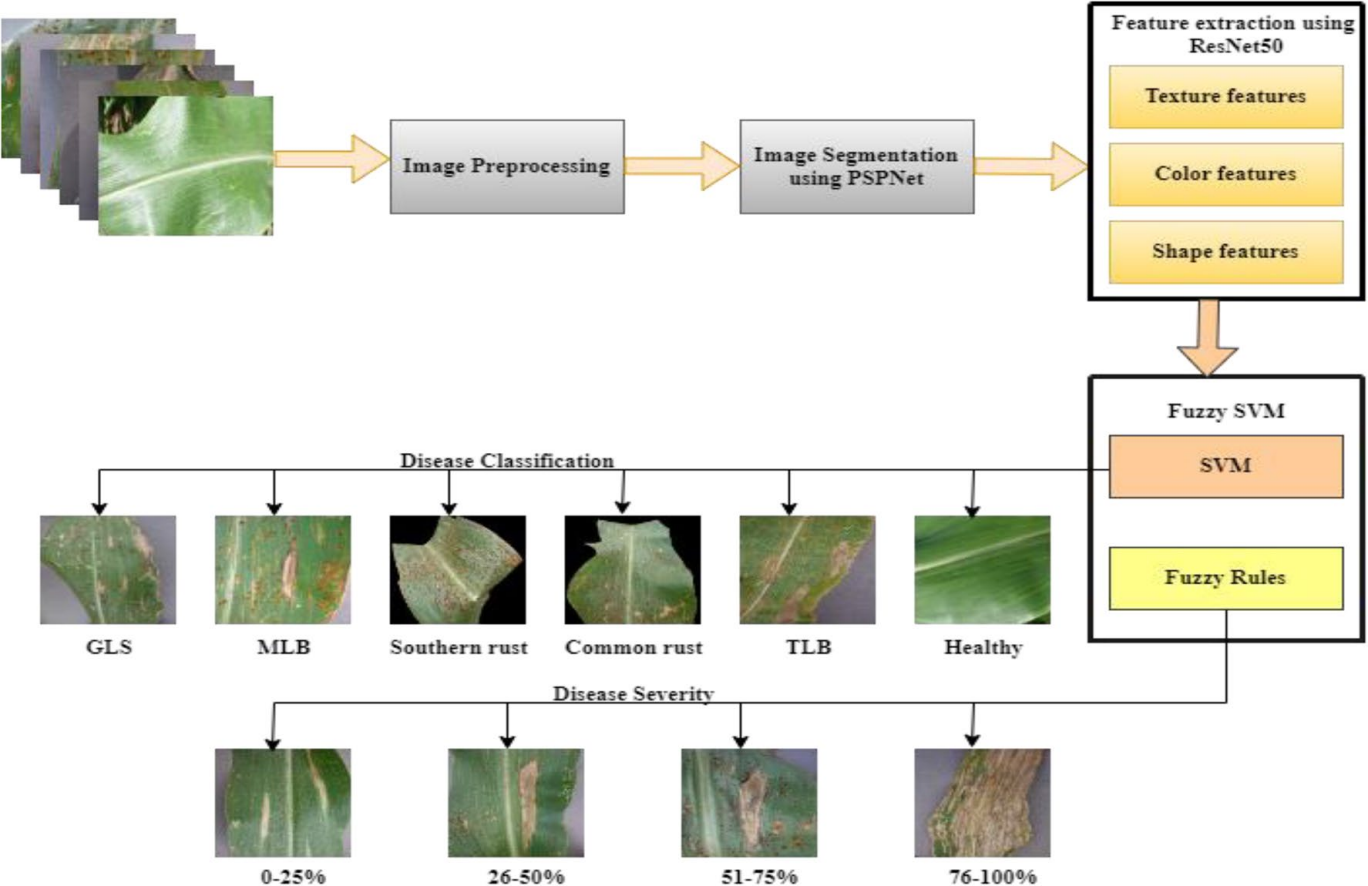
Proposed model.

This paper presents a proposed model viz. PRFSVM by combining three powerful components: PSPNet, ResNet50, and Fuzzy Support Vector Machine (Fuzzy SVM) as shown in Fig. 7. The goal of this paper is to push the boundaries of semantic segmentation by combining the strengths of these individual components to produce a highly effective and resilient image analysis system. To begin, the PSPNet (Pyramid Scene Parsing Network) architecture, is well-known for its superior performance in high-level image interpretation applications. PSPNet employs a pyramid pooling module to collect contextual information at several scales, allowing the model to produce pixel-level predictions with a richer context, improving semantic segmentation accuracy. In addition, the ResNet50 ^48^, which is well-known for its deep and residual design, extracts rich and hierarchical characteristics from input images. The combination of PSPNet and ResNet50 not only assures that the model can capture delicate visual features but also allows for end-to-end training for smooth integration. Fuzzy SVM is included as a final classification layer to accommodate the inherent fuzziness and uncertainty in real-world image data. Because Fuzzy SVM allows for soft categorization, that model can gracefully manage ambiguous and overlapping object boundaries. Fuzzy SVM's capacity to handle classification as well as severity evaluation. The advantages, limitations and challenges faced by proposed model have been described in Table 2.

**Table 2:** Perspectives of the proposed model.

Model	Advantages	Challenges	Limitations
PSPNet	Helps to recognize different objects of an image in a more effective manner^49^	Increased segmented occulted regions, which increases computing complexity^43^	Identify complex items in an image^27^
ResNet50	No training is required^20^	Compatible with each segmented region dimensions^22^	Skip connections between network layer^23^
Fuzzy SVM	Easy to construct fuzzy rules with different capabilities which may decrease the computational cost of the classification model^47^	Sensitive to features, making it challenging to define fuzzy rules in SVM models^13^	In cases of high complexity, this decision is done nonlinearly^47^

**Algorithm 1:**
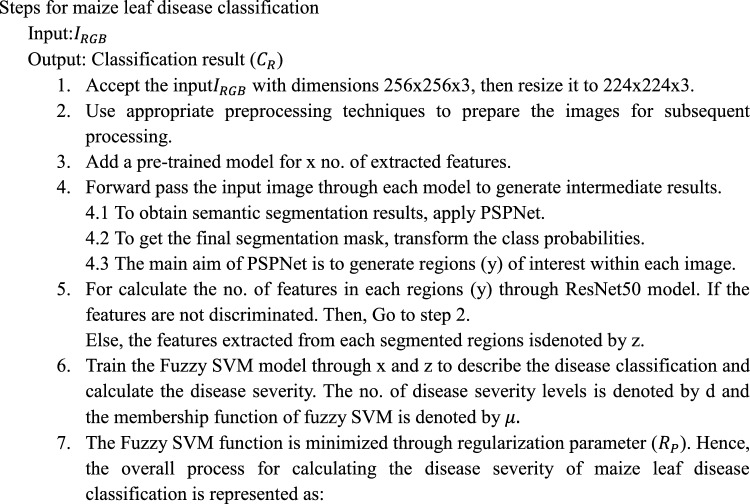
Proposed PRFSVM Model.

11 $${R}_{P}=\frac{1}{2} [ {\left|\left|W\right|\right|}^{2}+ {C}^{*}\sum \sum \mu \left(y\right)*\sum \sum \mu \left(z\right)*P(x,y,z)$$

12 $$P\left(x,y,z\right)={\text{x}}*{\text{z}}*({\text{W}}*\mathrm{\varnothing }\left(x,y\right)+c)\ge 1$$

### Navigating challenges and innovations: enhancing model synergy in a novel approach

- Integrated the three models namely PSPNet, ResNet50 and Fuzzy SVM model, activated the layers with different input and output dimensions.
- The segmentation and feature extraction models, extracts the features with different image resolutions. Thus, the combination approach of PSPNet and ResNet50 model ensures that it handle complementary capabilities of each network layer.
- Use feature alignment and normalization techniques to ensure that features from various components are in a similar space. This may include layer-wise normalization or feature scaling to improve the combined model's synergy.

## Experiment results, analysis and discussion

This section describes the implementation parameters in depth and numerous experiments were carried out to validate the suggested model's results. We thoroughly tested the proposed model's performance in terms of maize disease categorization and compared it to other networks using the maize leaf disease data set.

### Experimental setup and implementation details

The experiments of the proposed model were performed on an Ubuntu server 18.04 powered DELLEMC Power Edge R840 four-way rack server with an Intel Xeon(R) Gold 5120 processor and an Nvidia Tesla P100 GPU. The quality of images has been performed via the Gaussian filter on the Matlab 2019a version. The suggested PRFSVM model is implemented in Python using the TensorFlow and Keras frameworks. We used the pre-trained model on the collected dataset, which was trained on maize using transfer learning approach. The network is trained by using different hyper parameters such as batch sizes, learning rates, and the number of epochs with the Stochastic Gradient Descent (SGD) training optimizer to achieve the best results. The Hyper-parameter details of all parameters used in proposed model has been shown in Table 3.

**Table 3:** Hyper-parameter training parameters for the proposed model.

Parameter	Characteristics	Value
Batch size	If we tuning the batch size value, the grouping of each trained value increases the efficiency of the model	16
Epochs	Easy to learn underlying different patterns	50
Learning rate	Decreases the computational efficiency of layers	0.001
Minimum Threshold value for IoU	Helps to detect the segmented regions with no overlapping	0.5
Optimizer	Minimize the loss in segmentation regions	Adam
Weight decay rate	Increases the learning rate	0.005

### Performance parameters

The performance of the proposed model for maize disease classification and its severity is calculated through different performance parameters. For each class, we calculated the precision score (${P}_{S}$), recall score ${(R}_{S}$), accuracy (Acc), F1-score (${F}_{S}$), IoU score, and mean average precision (mAP) score parameters.

13 $${P}_{S} =\frac{{T}_{P}}{{T}_{P}+ {F}_{P}}$$

14 $${R}_{S}=\frac{{T}_{P}}{{T}_{P}+ {F}_{N}}$$

15 $$Acc=\frac{{T}_{P}+ {T}_{N}}{{T}_{P}+ {T}_{N }+{F}_{P}+ {F}_{N}}$$

16 $${F}_{S}= \frac{{{P}_{S}*R}_{S}}{{P}_{S}+{R}_{S}}* 2$$

Here ${T}_{P}$ means the true positive score, which represents the total number of positive samples that were correctly categorized as belonging to the target disease class. The ${F}_{P}$ is a false positive score that shows the total number of negative samples that were predicted to be positive. The ${F}_{N}$ stands for false negative, suggesting that the number of positive samples in the illness class was assessed inaccurately. Finally, ${T}_{N}$ signifies true negative samples, or those for which the model correctly predicted the negative class. For each class i, IoU is the intersection area of ​​the predicted bounding box and the ground truth bounding box in pixels, calculated using (17). On the other hand, mAP is the average of the average accuracy scores (AP) obtained for each i at different IoU threshold levels.

17 $${I}_{O}U= \frac{{T}_{Pi}}{{F}_{Pi}+ {F}_{Ni}+ {T}_{Pi}}$$

18 $$\mathrm{mAP }=\frac{1}{n}\sum_{i=1}^{n}{AP}_{i}$$

### Classification results of the proposed model

The classification results of the proposed model illustrate its efficacy in accurately categorizing data into predetermined classes or categories. The model achieves a high level of precision, recall, and F1-score through a mix of advanced deep learning techniques and robust training, suggesting its ability to properly detect and categories occurrences within the dataset. Furthermore, the model's performance is constant across multiple evaluation metrics, confirming its dependability and generalization capabilities. These categorization findings highlight the model's ability to solve real-world challenges and generate data-driven decisions, ultimately contributing to enhanced decision-making processes and outcomes in a variety of applications.

#### Segmented Sub-regions formed by PSPNet model

**Figure 8:**
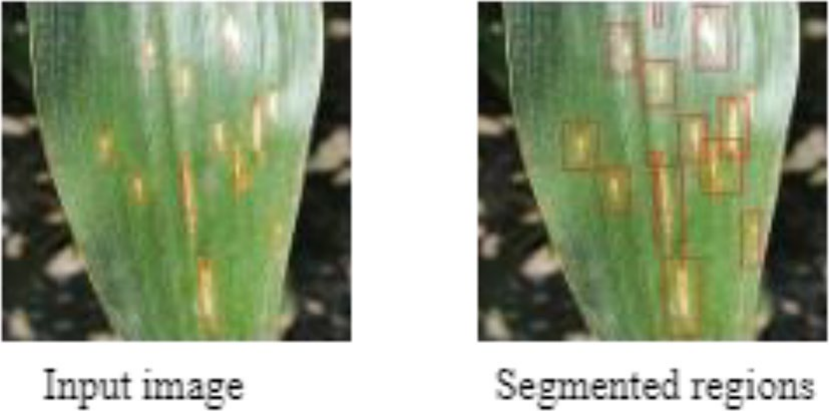
Segmented regions extracted by PSPNet models.

PSPNet is an image segmentation and labelling model that divides images into discrete sub-regions based on semantic value as shown in Fig. 8. This segmentation procedure entails breaking down a picture into fine-grained parts and labelling each segment uniquely based on its category or class. PSPNet captures multi-scale contextual information using a pyramid pooling module, which aids it in understanding the context and linkages between objects and regions within an image.

#### Features formulation

In this part, image feature extraction is done using a series of layers. It often consists of a number of convolutional and activation layers, followed by an integration layer. This step takes the pixel values ​​of the image as input and creates a feature map that is the input to the classification component. Convolutional layers consist of filters applied to the layer's input, allowing it to learn low-level, mid-level, and high-level visual features such as borders, texture, and color.

#### Classification and severity estimation

**Figure 9:**
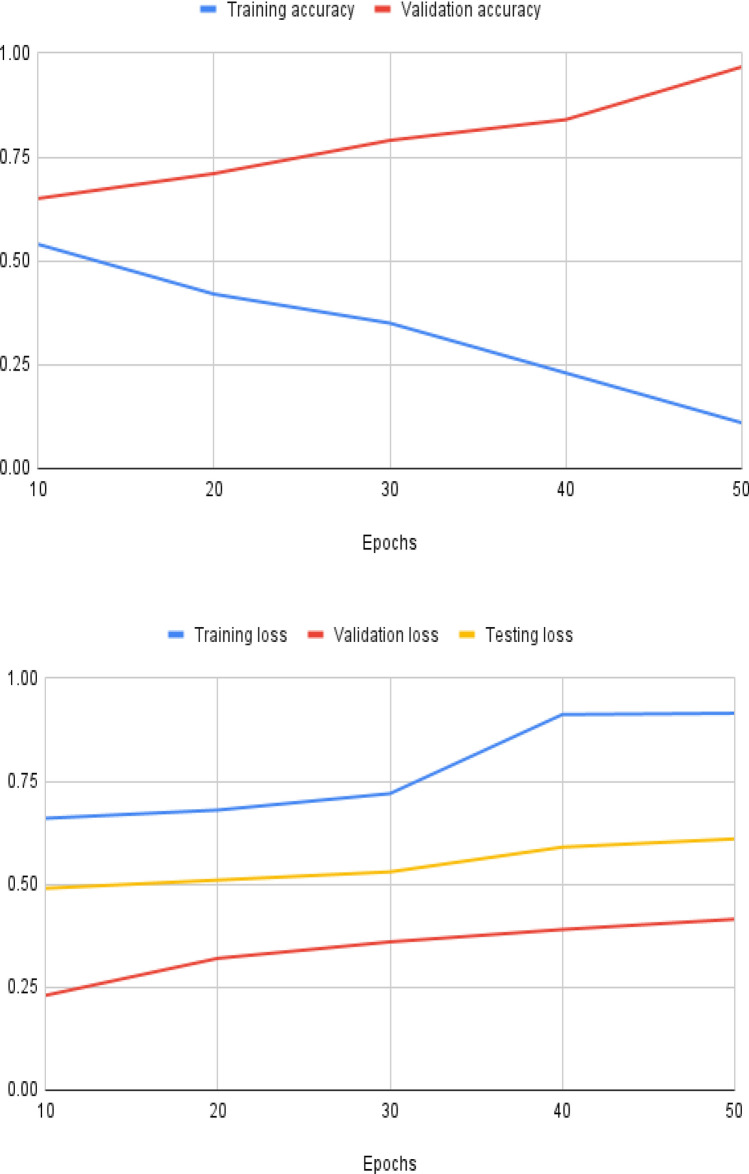
Training and validation loss/accuracy with different number of epochs.

In the field of data analysis and machine learning, classification and severity estimation are critical tasks. The purpose of classification is to assign data points to specified groups or classes, which allows for efficient organization and decision-making based on distinguishing features or attributes. Severity estimate, on the other hand, entails estimating the amount or level of a specific characteristic or condition inside a dataset. It offers a graded view of the facts, allowing for a more nuanced comprehension of circumstances. Classification and severity estimates work together to form the foundation of many machine learning and data analysis activities, allowing systems to categorize, prioritize, and respond to data in accordance with specified objectives and criteria. Accurate classification of diverse lesions is important for automatic detection of maize diseases using real-time field images. We reported the classification results of the proposed model for six major maize diseases using computational metrics such as precision, recall, f1 score and precision, recall, and f1 score. And the accuracy of this part. Table 4 displays the numerical evaluation results of the proposed model on each class. Each row represents a different class, while the columns indicate different performance criteria. The model achieves a high precision score of 0.877 for the MLB class, significance that it is correct within 87.7% of the time when predicting MLB. However, the recall score of 0.684 indicates that it fails to include all real MLB cases, skipping about 31.6% of them. The F1-Score of 0.789 provides a combined measure of accuracy by balancing precision and recall. For this class, the model has a remarkable overall accuracy of 0.956. The precision of the model for Southern Rust is 0.859, indicating that it accurately classifies this class with good accuracy. The recall score of 0.730, on the other hand, shows that some true Southern Rust instances are missed. The model obtains an overall accuracy of 0.937 for this class with an F1-Score of 0.763. The TLB class has a high precision (0.892), indicating that it is correctly identified. The recall score of 0.793 indicates that genuine TLB cases are covered rather well, but there is space for improvement. The F1-score for this class is 0.772, and the total accuracy is 0.952. For Common Rust, the model has high precision (0.891) and recall (0.810), showing great performance in detecting this class. The F1-Score is 0.804, indicating an excellent mix of precision and recall. At 0.967, the total accuracy is fairly high. GLS has a precision of 0.791 and a recall of 0.764, indicating a reasonable performance. The F1-Score is 0.737, indicating a good mix of precision and recall. The total precision is 0.941. The precision of the model for the Healthy class is 0.757, indicating occasional false positives. The recall score is 0.777, indicating that the majority of healthy situations are properly identified. The F1-Score for this class is 0.782, and the overall accuracy is 0.940. The overall performance of PRFSVM model has been shown in Table 2. Even, the training accuracy and validation accuracy with different number of epochs has been shown in Fig. 9. The severity of maize leaf diseases has been calculated through fuzzy rules. The fuzzy rules use the fuzzification and defuzzification rules for the calculation of disease severity. The severity is calculated in terms of disease infection level or macro disease index. Three different fuzzy rules has been making on the basis of their severity. The rules are defined as:

**Table 4:** Quantitative evaluation of the PRFSVM model's performance for each class.

Disease class	Precision	Recall	F1-Score	FNR	Accuracy
MLB	0.877	0.684	0.789	14.3	0.956
Southern Rust	0.859	0.730	0.763	10.2	0.937
TLB	0.892	0.793	0.772	11.5	0.952
Comon Rust	0.891	0.810	0.804	0.9	0.967
GLS	0.791	0.764	0.737	11.3	0.941
Healthy	0.757	0.777	0.782	12.1	0.940

- Severity is Low if the Disease Area is small and the lesion density is low. The term "Disease Area" refers to the percentage of leaf area damaged by lesions (from 0 to 100%). The term "Lesion Density" refers to the number of lesions per unit area.
- If the disease area or lesion density is moderate, the Severity is Medium.
- Severity is High if the disease area is large and the lesion density is high. If both the disease area and the lesion density are large, it indicates that the disease has widely impacted the leaf and caused severe damage.

**Figure 10:**
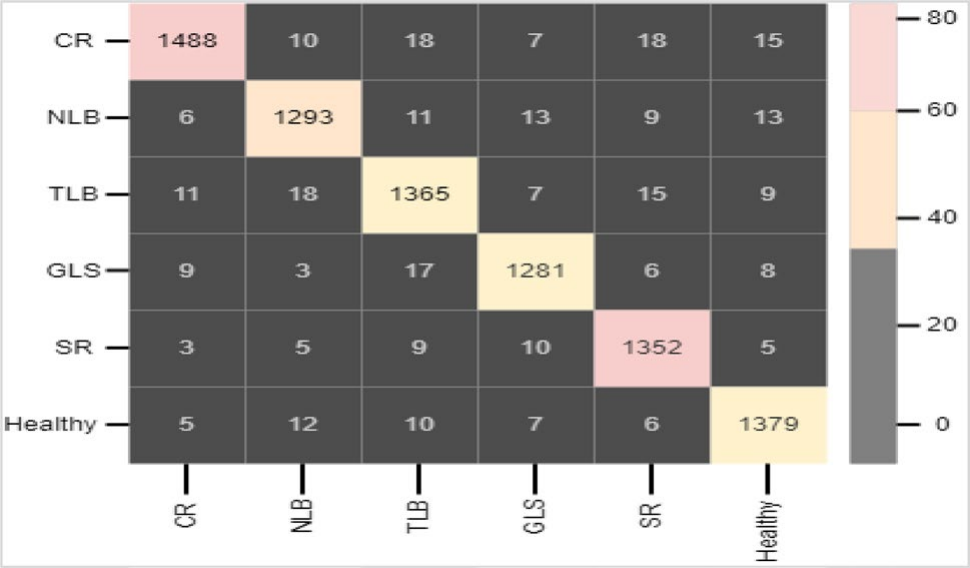
Performance of proposed approach: horizontal axis shows the predicted label and vertical axis shows the truth label for maize leaf diseases.

As a result, the severity is rated as "High”. These fuzzy rules take into account two crucial factors: the size of the disease-affected area and the density of lesions. To give additional specificity in your severity estimation, you can further fine-tune these criteria by creating particular linguistic phrases (e.g., "Very Low," "Very High") and membership functions for "Low," "Medium," and "High" severity. Figure 10 shows the performance of proposed approach i.e. horizontal axis shows the predicted label and vertical axis shows the truth label for maize leaf diseases.

## Discussion

This proposed model viz. PRFSVM by combining three powerful components i.e. PSPNet, RESNet-50, and Fuzzy SVM that have significant advantages in a variety of machine learning applications. PSPNet excels at semantic segmentation tasks, delivering precise object boundaries and semantic image understanding. With its deep architecture, RESNet-50 is highly effective for feature extraction and image identification, especially when huge amounts of data are available. Fuzzy SVM, on the other hand, is useful for dealing with imprecise and non-linear data, making it appropriate for classification in scenarios with ambiguous or complex connections between features. The ability of Fuzzy SVM to handle classification as well as severity evaluation.The combination of multiple models may improve classification accuracy and durability. The ROC curve shows the range between true positive rate (sensitivity) and false positive rate (1-specificity) for the PRFSVM model at different threshold levels.

**Figure 11:**
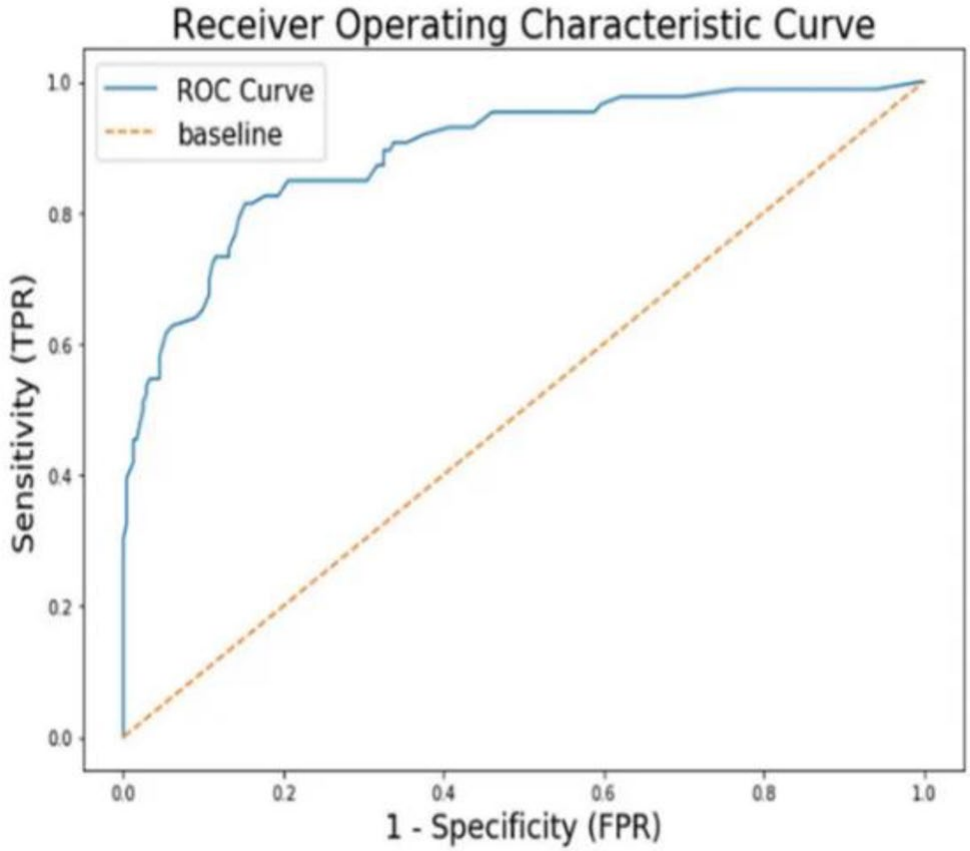
Precision, recall curve for maize leaf diseases identification.

The area under the ROC curve (AUC) measures the combined model's overall discriminative power. A well-separated ROC curve demonstrates that the combination of these models can produce high true positive rates while minimizing false positives. The combined method, as indicated in the Fig. 11, may offer enhanced resilience against distinct variances in disease severity presentations and can potentially manage a broader range of disease classes well. The multiple maize leaf diseases severity has been calculated through fuzzy rules. The fuzzy rules has been effectively calculated the severity of each disease in terms of low, medium and high levels.

To improve flexibility, the PRF-SVM model should be trained and tested on a variety of datasets, including maize varieties, geographies, and environmental circumstances. Experiments with different dataset sizes and computational resources may be used to illustrate the model's efficiency and scalability. The PRFSVM model was executed in 38.01 s. The proposed selection strategy outperforms a single classifier in terms of both execution time and accuracy.

### State of the art comparison

The Table 5 compares the new model to existing models. This research^17^ suggested a system for automatically detecting digital photos based on deep convolutional neural networks. From experimental fields in one, two, and three different maize growth zones, 5939 pictures of Maydis Leaf Blight, Turcicum Leaf Blight, and Banded Leaf and Sheath Blight were gathered. The maize dataset was used to model three network topologies for the 'Inception-v3' network. In a separate test dataset, Inception-v3_GAP had the highest accuracy of 95.99%. The paper^24^ proposed method comprises replacing the ResNet-50 backbone network's 3 × 3 convolution kernel with the Select Kernel-Point-Swish_B (SKPS) module, an upgraded feature derived from the chosen kernel (SK) unit. Furthermore, the typical ReLU activation function is substituted by the Swish_B activation function. These changes are intended to improve the model's ability to extract features from leaves with minor flaws and irregular forms, particularly those affected by diseases. The researchers^25^ evaluate the generalisation capacities of deep learning models when applied to the recognition of plant diseases in maize leaves, taking into account different datasets and variable environmental conditions. The study makes use of five unique datasets that contain images of foliar diseases in maize. Using various combinations of these datasets, many Deep learning (DL)based image categorization models were trained and evaluated. Transfer learning was implemented across four different experimental configurations using five different pre-trained deep neural network architectures: InceptionV3, ResNet50, VGG16, DenseNet169, and Xception.Notably, in all studies, the DenseNet169 model achieved higher accuracy. The paper^27^ used the ‘Simple Linear Iterative Clustering’ (SLIC) segmentation approach to analyse maize leaf pictures from both the CD&S and PlantVillage datasets. This method was used to create super-pixels, which are groups of pixels that represent different areas of interest on a maize leaf. Our proposed PRFSVM model is introduced, which combines the strengths of three main components: PSPNet, ResNet50, and Fuzzy Support Vector Machine (Fuzzy SVM). The model captures fine-grained visual information while also allowing for end-to-end training, providing smooth integration by combining PSPNet with ResNet50. As the final classification layer, Fuzzy SVM is used to account for the inherent uncertainty and fuzziness observed in real-world image data. The PRFSVM model has an average accuracy rate of 96.67%.

**Table 5:** Comparison with existing models.

References	Target	Datasets	Segmentation	Classification model	Recognition rate (%)
^17^	Maydis Leaf Blight, Turcicum Leaf Blight and Banded Leaf and Sheath Blight	Primary dataset (ICAR-IIMR, Ludhiana)	No	InceptionV3_GAP	95.99
^24^	Common rust, Southern, Maydis Leaf Blight, Turcicum Leaf Blight	Primary dataset (Maize fields in Hongyang,China)	No	ResNet50	92.90
^25^	Northern leaf blight, Gray leaf spot, common rust, Healthy and Northern leaf spot	PlantVillage, PlantDoc, Digipathos, NLB Dataset, CD&S Dataset	No	DenseNet169	81.60
^27^	Healthy, Northern leaf blight, Gray leaf spot, common rust	PlantVillage and CD&S Dataset	Yes	ResNet50	94.52
^50^	Healthy, gray leaf spot, blight, and common rust	Primary dataset (Farmers' fields in the Madura Region)	No	ResNet-50	95.59
Proposed model	Maydis Leaf Blight, Turcicum Leaf Blight, Gray leaf spot, common rust, Southern rust and Healthy	PlantVillage	Yes	Fuzzy SVM	96.67

### Limitations of the proposed model

- PSPNET typically cannot identify items in difficulty in low-resolution images of maize plants. The PRFSVM model's training speed drops after the planned mask is extracted from the maize plant image, increasing the computational cost of the model.
- By including fuzzy rules in the model parameters, the current study closes the gap between the newly trained model parameters and the original domain model, hence improving the transfer learning approach for small samples

### Practical implications of the proposed approach

The implications of proposed approach, particularly focused on maize leaf disease control and crop management, can have far-reaching consequences for farmers and other stakeholders in the agriculture industry. Here are some possible practical applications and implications:

- Early Disease Detection: The PRFSVM approach for detecting diseases in crops might greatly benefit farmers. Early detection allows for timely action, which reduces disease transmission and crop losses.
- Precision agriculture involves integrating advanced technologies. Farmers can utilize innovative approaches to customize their interventions, such as spraying pesticides or fertilizers, to the precise needs of individual plants or areas of the field. 3. Decreased maize yield quality: Breakthrough strategies boost agricultural output and quality by efficiently managing diseases. Healthy crops are more likely to attain their full potential, increasing farmers' productivity.
- Minimize Environmental impact: Targeted interventions using innovative techniques can reduce the usage of agrochemicals, leading to a lower environmental impact. This not only saves farmers money, but it also reduces the environmental impact of using too much pesticide or fertilizer.

## Conclusions and future directions

Diseases that affect maize leaves cause a substantial risk to maize harvests all over the world, resulting in decreased yield and economic losses. There are several fungal diseases, including like common rust, southern rust, MLB, TLB and gray leaf spot. To decreases the maize diseases quality yield losses, proposed approach namely as PRFSVM is proposed in this paper. The proposed approach consists of PSPNET, ResNet-50, and Fuzzy SVM that estimates the three different classes of Maize diseases along with their severity. The PSPNET and ResNet-50 neural networks exhibited their ability to identify and segment maize leaf diseases from images. The combination of these deep learning models aided in achieving a high level of disease recognition accuracy (96.67%) as compared to previous studies. With the usage of Fuzzy SVM model in proposed approach, it estimates the disease severity. Even, the proposed approach can be linked into precision farming systems to give real-time disease monitoring, allowing for prompt interventions and decreasing crop losses. We proved the possibility for precise and practical disease control in agriculture by combining PSPNET, ResNet-50, and Fuzzy SVM. This research advances agricultural technology and has the potential to considerably benefit farmers by ensuring crop health and increasing yield. The present research discussed the DL techniques for detecting several maize leaf diseases. Additionally, a summary of visualisation approaches and mappings was provided to identify the maize leaf disease symptoms. While tremendous progress has been made, there are certain research gaps, as stated below.

- Several studies used the PlantVillage dataset to assess the precision and effectiveness of DL models and architectures. Whereas this dataset contains many images of various plant species and their diseases, it includes a simple/plain environment.
- To ensure an efficient DL model, datasets should include photographs from several field situations in addition to the real environment.
- Introducing a more efficient technique to visualise disease spots in maize leaves can save money by reducing the need for excessive pesticides, chemical, and insecticide applications.
- In future, the proposed approach is implemented in real time IoT device so that the potential yield maize loss is decreased.

Paving the path forward: future research and development in Maize leaf diseases recognition alongside emerging trends

- The secondary sources dataset of Maize leaf disease are frequently small in size, and obtaining large amounts of labeled data is resource-intensive.
- This technique has the potential to reduce data scarcity while also improving the model's capacity to generalize across different diseases kinds and environmental variables.
- Ensemble models, such as stacking or boosting, may improve overall classification performance by merging the distinct information gathered by each model.

## Data Availability

The data supporting the research results of the present research can be obtained by contacting the corresponding author.
